# An economic evaluation of first-line cryoballoon ablation versus antiarrhythmic drug therapy for the treatment of paroxysmal atrial fibrillation from a German healthcare payer perspective

**DOI:** 10.1186/s12913-024-11967-0

**Published:** 2024-11-26

**Authors:** Malte Kuniss, Lucy Hillcoat, Joe Moss, Florian Straube, Jason Andrade, Oussama Wazni, Gian Battista Chierchia, Lukas Schwegmann, Eleni Ismyrloglou, Alicia Sale, Stuart Mealing, Tom Bromilow, Emily Lane, Damian Lewis, Andreas Goette

**Affiliations:** 1 Kerckhoff Heart Center, Bad Nauheim, Germany; 2 York Health Economics Consortium, York, UK; 3 Heart Center Munich-Bogenhausen, Munich, Germany; 4 University of British Columbia, Vancouver, BC Canada; 5 Cleveland Clinic, Cleveland, OH USA; 6 Universitair Ziekenhuis Brussel and Vrije Universiteit Brussel, Brussels, Belgium; 7 Medtronic, Meerbusch, Germany; 8 Bakken Research Center B.V, Maastricht, Netherlands; 9 Medtronic, Mounds View, Minnesota USA; 10 Vincenz Hospital, Paderborn, Germany

**Keywords:** Ablation, Cryoablation, Cost-effectiveness, Paroxysmal atrial fibrillation, Antiarrhythmic drug

## Abstract

**Background:**

Three recent randomized controlled trials demonstrated that, in patients with symptomatic paroxysmal atrial fibrillation (PAF), first-line pulmonary vein isolation with cryoballoon catheter ablation reduces atrial arrhythmia recurrence compared to initial antiarrhythmic drug (AAD) therapy. This study aimed to evaluate the cost-effectiveness of first-line cryoablation compared to first-line AADs from a German healthcare payer perspective.

**Methods:**

Individual patient-level data from 703 participants with untreated PAF enrolled into three randomized clinical trials (Cryo-FIRST, STOP AF First and EARLY-AF) were used to derive parameters for the cost-effectiveness model (CEM).

The CEM structure consisted of a hybrid decision tree and Markov model. The decision tree (one-year time horizon) informed initial health state allocation in the first cycle of the Markov model (40-year time horizon; three-month cycle length). Health benefits were expressed in quality-adjusted life years (QALYs). Cost inputs were sourced from German diagnosis-related groups and the Institute for the Hospital Remuneration System (InEK). Costs and benefits were discounted at 3% per annum.

**Results:**

Cryoablation was cost-effective, incurring ~ €200 per patient while offering an increase in QALYs (~ 0.18) over a lifetime. This produced an average incremental cost-effectiveness ratio of ~ €1,000 per QALY gained. Individuals were expected to receive ~ 1.2 ablations over a lifetime, regardless of initial treatment. However, those initially treated with cryoablation as opposed to AADs experience 0.9 fewer re-ablations and a 45% reduction in time spent in AF health states.

**Conclusion:**

Initial rhythm control with cryoballoon ablation in symptomatic PAF is a cost-effective treatment option in a German healthcare setting.

**Supplementary Information:**

The online version contains supplementary material available at 10.1186/s12913-024-11967-0.

## Background

Atrial fibrillation (AF) is the most common form of cardiac arrhythmia, with a worldwide prevalence of approximately 37 million cases [1]. Although pathology may differ between individuals, symptoms frequently include light-headedness, shortness of breath, fatigue, and heart palpitations [2]. Paroxysmal AF (PAF) is an episodic variant of AF that terminates spontaneously or with intervention within seven days of onset and is estimated to comprise between 25 to 65% of all AF cases [3, 4].

AF is associated with an increased risk of adverse health outcomes, including ischemic stroke, heart failure and myocardial infarction, cognitive impairment, and mortality [5, 6]. If symptoms continue beyond seven days, PAF can develop into a more sustained condition, such as persistent, long-standing persistent or permanent AF, which increases the risk of negative cardiovascular outcomes [6]. Consequently, AF is associated with reduced health-related quality of life (HRQoL) and rising healthcare costs [5, 6]. The economic impact of AF in Germany is substantial, with an annual cost of over €960 million incurred to the statutory health funds (estimated 2023 values) [7].

In Germany and according to the latest ESC 2020 guidelines [4] first-line catheter ablation (CA) for AF should be considered (Class IIa) and may be considered (Class IIb) for treating symptomatic patients with paroxysmal and persistent AF. Although the class of recommendation remained unchanged, a significant increase in CA as a first-line treatment for PAF in symptomatic patients without structural heart disease PAF has been reported [8]. Currently, six randomized clinical trials utilizing either radiofrequency or cryoballoon catheters have demonstrated the superiority of CA for AF as a first-line treatment over medical therapy [9–14].

Furthermore, ablation is recommended, in general, as a second-line therapy after failure (or intolerance) of class I or class III antiarrhythmic drug (AAD) [6]. During pulmonary vein isolation (PVI), a method of ablation, catheters are used to deliver hot or cold temperatures within the left atrium to scar or destroy tissue around pulmonary veins (PVs) that cause irregular atrial contractions. Durable electrical disconnection of the PVs from the left atrium is the cornerstone of AF ablation. Cryoballoon ablation (“cryoablation”) is a method of PVI that uses a single-delivery approach where cryothermia is applied in a balloon catheter to freeze tissue [7].

Three recent randomized control trials (RCTs) investigated whether first-line cryoablation is the optimal initial rhythm control technique for patients who are not intolerant or refractory to AADs; Cryo-FIRST (NCT01803438, registration date 2013–03-01), STOP AF First (NCT03118518, registration date 2017–04-04), and EARLY-AF (NCT02825979, registration date 2016–05–18) [9, 10, 15]. These trials assessed the superiority of cryoablation over AADs for preventing atrial arrhythmia recurrence. The RCTs enrolled 703 participants with symptomatic PAF who were randomized (1:1) into two initial treatment arms (cryoablation and AADs). The findings showed that cryoablation is superior to AADs for reducing arrhythmia recurrence as an initial rhythm control strategy. Additionally, cryoablation was associated with a low rate of device- or procedure-related serious adverse events (AEs). Moreover, first-line cryoablation versus AADs was associated with a lower incidence of progression to persistent AF over three years [15].

Previous research by McBride et al. [7] on the costs associated with AF in Germany recommended a focus on the avoidance of AF hospital admission, stroke prevention and rhythm control optimisation to reduce the economic burden of AF. Due to the potential benefits of first-line cryoablation identified in the primary clinical trials and the conclusions of McBride et al. [7], this study investigated the use of first-line cryoablation from a German healthcare payer perspective using data generated by the Artic Front Advance cryoablation randomized clinical trials [9, 10, 15]. The aim was to assess the impact of first-line cryoablation on both the overall economic burden of AF in Germany but also to conduct a cost-effectiveness analysis (CEA) to evaluate the impact of first-line cryoablation versus first-line AADs for treating symptomatic PAF. CEA is pertinent for decision making for novel treatments ahead of implementation into routine care and guidelines, to ensure the cost will be worth the subsequent benefits. Given the economic and HRQoL burden caused by AF in Germany, and the evidence from clinical trials illustrating cryoablation is superior as an initial rhythm control strategy, the current study evaluated the cost-effectiveness of first-line cryoablation versus first-line AADs for treating symptomatic PAF, with intent to support decision making surrounding implementation.

## Methods

### The economic model

The hybrid model consisted of a decision tree and Markov structure. In both model components, costs and health benefits were captured for a hypothetical cohort of 1,000 untreated symptomatic PAF individuals with a baseline age of 57.5 years old. Individuals were initially allocated a disease state reflective of the population in the three randomized clinical trials and detailed in Table S1 in the Supplementary Materials [9, 10, 15]. The current model was adapted from a global economic model, from which other adaptations have been published [16–19]. Inputs for the current cost-effectiveness model (CEM) were updated to reflect a German healthcare payer perspective, and costs and benefits each had a 3% discount rate per year applied [20]. Health benefits were expressed as quality-adjusted life years (QALYs).

The primary endpoint for the three RCTs that informed the analysis were assessed after 12 months. Therefore, to align with this, the decision tree at the beginning of the model had a one-year time horizon [9–11]. This component of the CEM was used to allocate patients to one of three health states, including and defined as: normal sinus rhythm (NSR—no AF recorded within three months); short-term (ST) episodic (at least one AF episode—paroxysmal or persistent—recorded within three months); and death. To align with the three-monthly cycle length applied in the subsequent Markov model, these health state definitions were used in place of conventional definitions. They are based on clinical definitions from the European Society of Cardiology [6] and were validated by the clinical authors (listed) to capture disease progression whilst reflecting clinical definitions.

The Markov model had a three-month cycle length, chosen to portray the recurring nature of PAF-associated arrhythmia throughout a year. Additionally, a 40-year life-time horizon was implemented to capture all cost and health outcomes associated with the model cohort. Whilst in the Markov model, patients were predicted to occupy one of five health states: NSR, ST episodic, long term (LT) persistent, permanent AF, or dead. Health states NSR, ST episodic, and LT persistent each had four substates (0–3) to represent the number of repeated procedures each patient had received, where more repeated procedures are associated with a corresponding relative risk (RR) of symptom recurrence or resolution. Patient movement between health states was derived from the statistical analysis of the individual patient data (IPD) from the three RCTs which captured long-term patient outcomes (Sect. 1 and 2 Supplementary Materials). A hypothetical willingness-to-pay (WTP) threshold of €35,000 per QALY gained was observed, due to Germany not having an official cost-effectiveness threshold. This boundary has been utilized within previous German cost-utility studies for cardiovascular disease conditions, mirroring the upper limit of the UK National Institute for Health and Care Excellence (NICE) threshold [21, 22].

In clinical practice today, clinical expert consensus statements recommend a 3-month post ablation “blanking period” [23]. This is a period in which AF recurrences are believed to be a result of transient inflammation, un-associated with later AF recurrence, and thus should not be deemed a treatment failure [24]. In the analysis, in order to capture the full healthcare utilization and associated costs, the initial 12-week data was included in the base case model. The effect that including a blanking period has on the results was explored through scenario analysis.

Within the decision tree model the number of ablations following initial procedure (“re-ablations”) was captured by an ablation count within the NSR and ST episodic health states. For both treatment arms, if patients received, for example, one re-ablation (exclusive of the initial procedure in the cryoablation arm) they were kept in numeric sub-state ‘1’ of the health state they occupied at the end of the decision-tree (e.g. ST-episodic 1). The patients’ final outcome within the decision tree determined their initial state allocation in the Markov model.

**Fig. 1:**
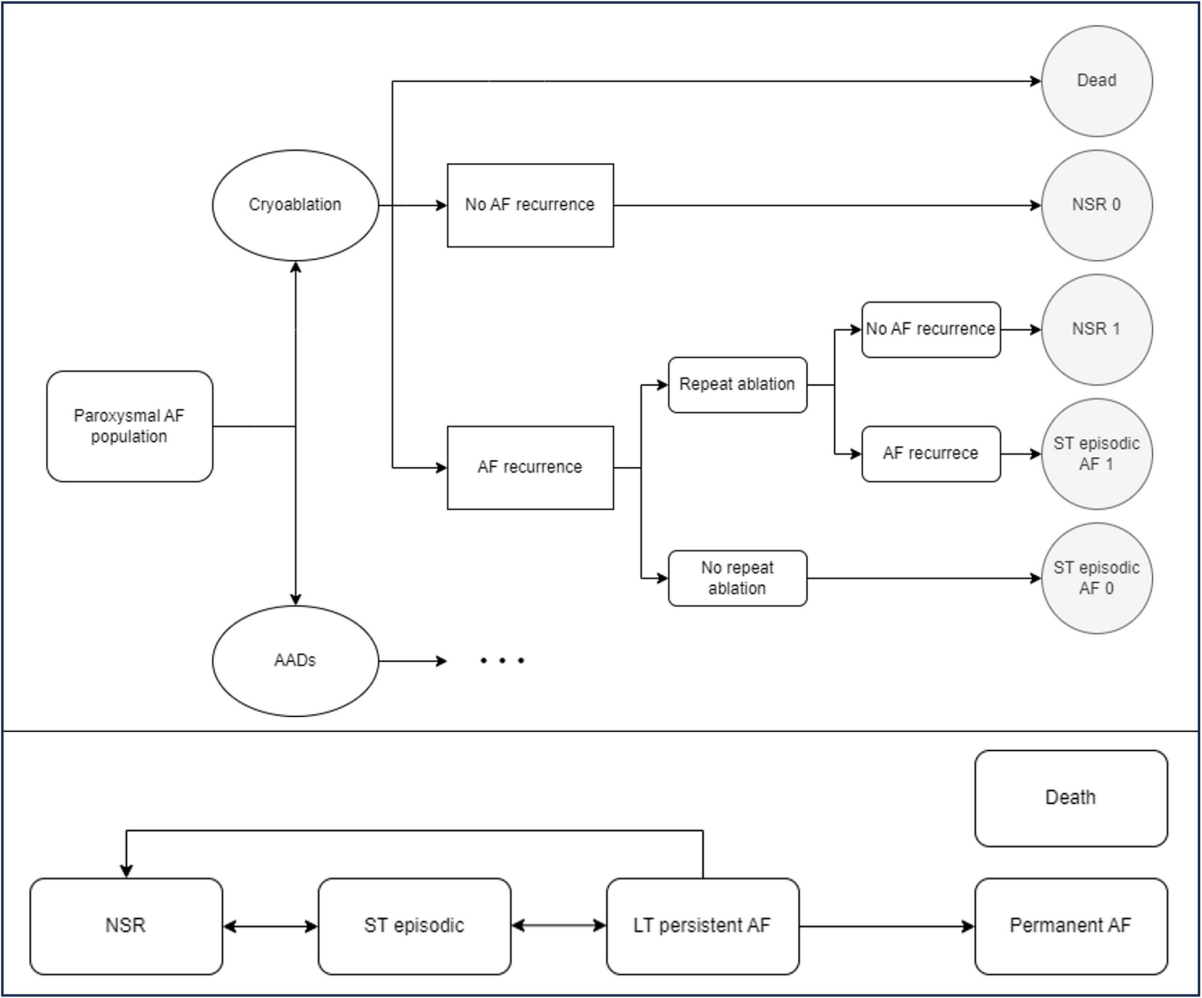
Schematic of the economic model: **a** Decision tree used to model the first 12 months of the economic evaluation, where the endpoints constitute initial Markov model health state allocation **b** Markov model used to model the remaining lifetime in the economic evaluation. NSR, ST episodic and LT persistent AF states are broken down into four subhealth states, from zero to three, indicating the number of re-ablations received (either one initial and two repeat ablations in the cryoablation arm or three repeat ablations in the AAD arm. The maximum number of possible total ablations in both arms was set to three). Death is permitted from all other health states. AF – Atrial Fibrillation, AADs – Antiarrhythmic Drugs, ST – Short term, NSR – Normal Sinus Rhythm, LT– Long Term

The Markov model component of the CEM incorporated two additional health states: LT persistent (AF symptoms that remain over at least a 12-month duration and do not remit without treatment), and permanent (AF where, accepted by the patient and physician, no further attempts to restore or maintain NSR will be undertaken). In line with the decision tree, numeric sub-health states were assigned to patients in the Markov model corresponding to the number of re-ablation procedures they received, excluding the initial procedure in the cryoablation arm. A maximum of three ablation procedures could be applied – including the initial procedure to align with real world practice. The model structure is presented in Fig. 1.

### Assumptions

Parameters in the model that were based on assumptions included: RR parameters for AF recurrence and resolution, stroke, heart failure, and re-ablation success according to the number of ablations received and health state occupied. These parameters were conservative estimates following validation of clinical plausibility by clinical experts. Likewise, the stroke rates included in the CEM were established following clinical opinion because suitable parameters were not identified in the literature. The utility decrement applied to the ST-episodic and LT-persistent states were assumed to be equivalent since it was unintuitive for LT-persistent to align with a better utility than ST-episodic. Extensive scenario analyses were conducted to explore parameter uncertainty and demonstrated that a cost-effective result was maintained across all scenarios explored, as presented in Table 5.

### Model parameters

IPD from 703 patients with symptomatic PAF who were enrolled into the Cryo-FIRST, STOP AF First and EARLY-AF clinical trials were used to derive prognostic equations, informing input parameters for the CEM. Whilst similar analyses have been undertaken in UK [16], United States of America (USA) [17], Canadian [18] and Danish [19] healthcare settings, the current paper focuses on the healthcare service perspective within Germany. To ensure transparency, the statistical methods and outcomes of the IPD analysis, including key model parameters such as detailed utility values, costs, event rates, and mortality calculations are described in full in Sect. 1 of the Supplementary Materials, Additional file 1.

The outcomes from the analyses of relevance to the CEM included: rate of outpatient appointments, rate of emergency department visits, rate of pharmaceutical and electrical cardioversion, rate of AF-related hospitalisation, rate of repeat ablation (re-ablation), AF recurrence and resolution, and EQ-5D-3L utility values.

Key model parameters are displayed in Table 1. Where possible, parameter estimates were derived from the IPD analyses. Where information was not collected in the RCTs, this was sourced by targeted literature searching for localised, high quality evidence using tools such as PubMed and Google Scholar. If the required information did not exist in the literature, the named clinical authors provided estimates for parameters. Clinical co-authors were interviewed until a consensus for all inputs was achieved and based on their clinical experience these inputs were considered both reasonable and conservative. The same panel of clinical experts also validated the structure of the economic model to ensure it was reflective of the clinical pathway. Costs and RR values were informed by a combination of literature searching and clinical consultation.

**Table 1:** Key model input parameters †

Parameter	Value ‡	Source
Unit Costs		
* Procedure-related cost*		
Ablation procedure	€8,121	[35, 36] Derived from aG-DRGs, InEK Fallpauschalenkatalog as described in Table S13
*Healthcare contact costs (per cycle)*		
CV-related hospitalizations (excluding re-ablation procedures)	€1,464	[35]
CV-related A&E department visits (excluding re-ablation procedures)	€32	[37]
CV-related outpatient appointments (excluding re-ablation procedures)	€107	[38]
Pharmaceutical cardioversion	€1,206	[39]
Electrical cardioversion	€166	
*Atrial fibrillation AE costs (per cycle)*		
Non-disabling stroke	€1,204	[36]
Moderately disabling stroke	€2,864	
Severely disabling stroke	€5,168	
Stroke long-term cost	€391	[40]
Heart failure (NYHA class I)	€166	[41]
Heart failure (NYHA class II)	€213	
Heart failure (NYHA class III)	€244	
Heart failure (NYHA class IV)	€291	
*Pharmaceutical costs (per cycle)*		
Cryoablation arm	€69	[42] Derived from per cycle pharmaceutical costs weighted by resource use at 12 months as described in Tables S12a and S12b
AAD Arm	€89	
Utility Decrements		
* Health state decrements*		
LT-persistent	0.08	Assumed to match the decrement for ST-Episodic derived from the clinical trial data
Permanent	0.11	Decrement of permanent AF versus paroxysmal AF derived from [5] and applied to the ST-Episodic decrement derived from the clinical trial data
*AE decrements*		
Non-disabling stroke – short-term	0.00	[43]
Moderately disabling stroke – short-term	0.23	
Severely disabling stroke – short-term	0.60	
Non-disabling stroke – long-term	0.00	
Moderately disabling stroke – long-term	0.17	
Severely disabling stroke – long-term	0.35	
Heart failure (NYHA class I) – long-term	0.00	[43]
Heart failure (NYHA class II) – long-term	0.05	[41]
Heart failure (NYHA class III) – long-term	0.15	
Heart failure (NYHA class IV) – long-term	0.33	

*Abbreviations:* *AAD* antiarrhythmic drug, *AE* adverse event, DRG Diagnosis Related Groups, *InEK* Institute for the Hospital Remuneration System, *LT* long-term, *ST* short-term

†The cited parameters include those that were not derived from the analysis of the individual patient-level data

‡ Where appropriate, costs were inflated to 2023 values

### Costs

Unit costs were sourced from German diagnosis-related groups (aG-DRGs), the Institute for the Hospital Remuneration System (InEK), the Rote Liste, and literature-sourced values (Table 1). Where appropriate, costs were inflated to 2023 values using a euro-specific inflation calculator [25]. Treatment costs were applied when patients entered the model and at any instance of a repeated ablation procedure. Additional costs such as pharmaceuticals, additional health care costs, and AE-related costs were also captured in the model and applied per cycle to the sum of individuals in each health state. The method used to calculate the ablation procedure unit cost is summarised in Supplementary Table S13, Additional file 1.

### Utility values

The impact of symptom severity and AEs on HRQoL was captured by applying disutility to baseline utility norm values. The baseline utility norms were weighted by sex according to the distribution identified from the pooled trial data (Table 1). The utility values applied in the current model were taken from the European EQ-5D-5L index time trade-off (TTO) value set, and was mapped to the EQ-5D-3L by means of the van Hout crosswalk function algorithm [26, 27]. Utilities were mapped to the EQ-5D-3L for the original global model that was built from a UK perspective in accordance with NICE preferences [28]. Hence, this was also done for the current, locally-adapted model.

### Adverse events

In all living health states, AE probabilities were applied to the population to reflect the likelihood of a patient having a stroke or heart failure. The probability of stroke was health state and age-dependent (Table S15 to S18) and based on the cohorts’ CHA₂DS₂-VASc score. The probability of heart failure was health state and age-dependent (Table S19 and S20) and based on the general population data.

### Mortality

Mortality was captured via a combination of German general population life tables (adjusted to exclude stroke- and heart failure-related deaths) and published stroke- and heart failure-related mortality rates (Tables S19 to S21). The mortality rates were weighted by sex using the proportion identified in the pooled clinical trial data. The formula used for the overall mortality rates is presented in Sect. 4 of the Supplementary Materials, Additional file 1. The final annual rates used were converted to three-monthly rates to align with the CEM.

### Probabilistic sensitivity analysis

A probabilistic sensitivity analysis (PSA) was conducted to assess the uncertainty of model parameters and results, as well as generate the mean cost and QALY outcomes per patient across 5,000 model iterations. Furthermore, the 95% credible intervals (CrI) either side of these mean values, the mean incremental cost-effectiveness ratio (ICER), and the probability of cryoablation being cost-effective were also reported. Distributions were fitted to uncertain parameters within the model to generate the inputs for each iteration. Gamma distributions were used for cost parameters and beta distributions were used for probability and utility parameters. Uncertainty regarding the estimates provided by the regression equations was incorporated into the model through using the Cholesky matrix derived from the regression variance–covariance matrix. Model parameters estimated by clinical experts were subject to an uncertainty level of 10%.

### Scenario analyses

Scenario analyses were conducted to explore parameter uncertainty, where input parameters were changed to those obtained from alternative sources or varied according to clinical expert opinion, or where a 12-week blanking period was applied. The following parameters were explored in the scenario analyses: AF recurrence and resolution risk, ablation success rate, stroke incidence rate, the health state-specific RR of stroke and the RR of heart failure in the permanent state. Ablation success following the procedure is determined by whether the patient returned to NSR for 90 day without experiencing further AF symptoms.

### Model outcomes

Common health economic outcomes are reported in the Results section, such as cost per patient, QALYs per patient, ICER, probability of being cost-effective. A series of scenario analyses to assess model robustness was also complete. Additional relevant outcomes, for example the incremental life-years gained per person, the time spent in AF health states and cost per stroke event avoided, have also been presented for further detail.

### Software

The economic model was constructed in Microsoft Excel.

### CHEERS checklist

A completed CHEERS checklist has been reported in the supplementary materials.

## Results

The PSA results indicate that the cryoablation arm is estimated to yield higher costs (+ €191 [95% CrI = -€1,367 to €1,745]) and QALYs (+ 0.18 QALYs [95% CrI = 0.04 to 0.38]) per person than the AAD arm over a lifetime horizon. Ultimately, a mean ICER of €1,037 (95% CrI = €392 to €23,065) per QALY gained was produced (Table 2).

**Table 2:** Probabilistic cost-effectiveness results (mean, 95% CrI)

Treatment	Cryoablation	AADs	Incremental
Cost (per patient)	€18,548 (€16,813 to €20,453)	€18,356 (€16,534 to €20,398)	€191 (-€1,367 to €1,745)
QALYs (per patient)	14.53 (14.323 to 14.707)	14.35 (13.979 to 14.648)	0.18 (0.04 to 0.38)
ICER			€1,037 (€392 to €23,065)
NMB			€6,271 (€911 to €13,341)
Probability of cost-effectiveness at a WTP threshold of €35,000 per QALY gained			98.0%

*Abbreviations:* *AADs* antiarrhythmic drugs, *CrI* credible interval, *ICER* incremental cost-effectiveness ratio, *NMB* net monetary benefit, *QALY* quality adjusted life-year, *WTP* willingness-to-pay

**Fig. 2:**
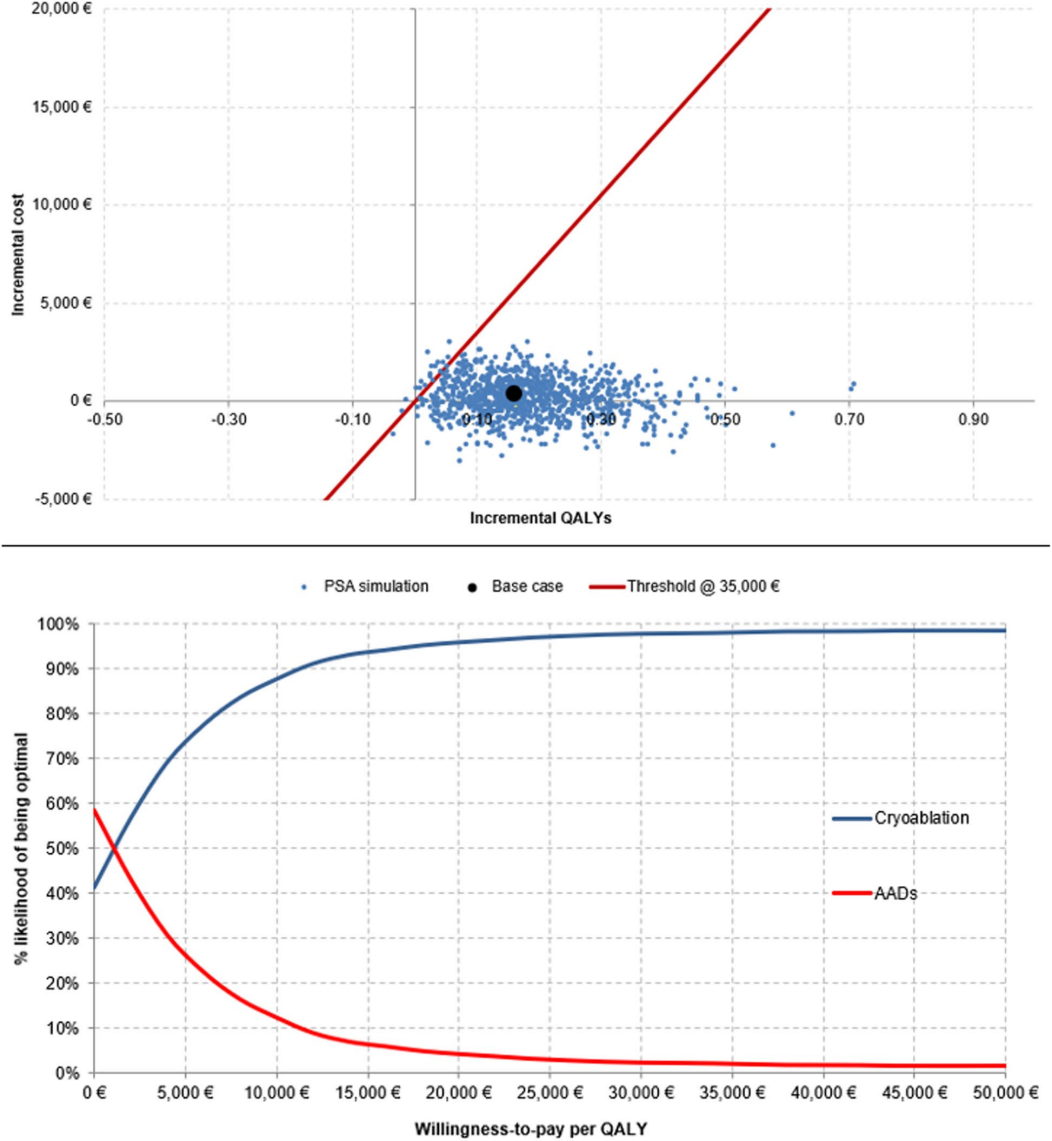
Probabilistic sensitivity analysis (PSA) graphical outputs: **a** Cost-effectiveness plane – Most iterations fell into the North-East quadrant below the €35,000 WTP threshold per QALY gained, signifying cryoablation is more effective and costlier than AADs, **b** Cost-acceptability curve – Illustrating the impact of uncertainty on results. The blue line indicates that cryoablation has 98.0% likelihood of being cost-effective at the WTP threshold

The majority of PSA iterations fell in the North-East quadrant of the cost-effectiveness plane (Fig. 2), signalling that cryoablation is more effective and costlier than AADs. The analysis indicated that cryoablation was cost-effective in 98.0% of iterations at a WTP threshold of €35,000 per QALY and cost saving in 41.5% of all simulations (Fig. 2).

A breakdown of the deterministic results illustrates that, although cryoablation has a costlier initial procedure, other cost outcomes such as re-ablations, healthcare contact costs and pharmaceutical costs are lower for cryoablation than AADs (Table 3).

**Table 3:** Breakdown of deterministic cost-effectiveness results (per patient)

Outcome	Cryoablation	AADs	Incremental
Initial procedure	€8,121	€0	€8,121
Re-ablations	€1,645	€6,998	-€5,352
Healthcare contact costs	€651	€1,618	-€967
Pharmaceutical costs	€4,656	€5,996	-€1,340
AF-related adverse events	€3,362	€3,464	-€102
Total cost per patient	€18,435	€18,075	€360
QALYs per patient	14.55	14.39	0.16
Incremental cost-effectiveness ratio (ICER)			€2,227

*Abbreviations:* *AADs* antiarrhythmic drugs, *AF* atrial fibrillation, *ICER* incremental cost-effectiveness ratio, *QALY* quality-adjusted life years

Table 4 presents additional results per patient. The cryoablation arm reported 0.04% higher predicted life years gained resulting in an ICER per life year gained of €48,564, and a 5.1% lower lifetime rate of stroke than the AADs arm. The cryoablation arm also demonstrated 76.5% fewer re-ablations received, and reduced time spent in AF health states (40.7%, 46.7%, 46.7% for ST-episodic, LT persistent and permanent, respectively) for those who received cryoablation compared to AADs.

**Table 4:** Additional deterministic model results (per patient)

Outcome	Cryoablation	AADs	Incremental	Relative change	Cost per event avoided	NNT
Time Spent in Each State (Years)						
Normal sinus rhythm	21.99	19.91	2.08	10.5%		
Short-term episodic	2.24	3.79	−1.54	−40.7%		
Long-term persistent	0.33	0.62	−0.29	−46.7%		
Permanent	0.27	0.51	−0.24	−46.7%		
Life Years						
Undiscounted life years	24.83	24.82	0.02	0.07%		
Discounted life years	16.77	16.77	0.01	0.04%		
Lifetime Adverse Event Rates						
Stroke	0.261	0.275	−0.01	−5.1%	-€25,897	72
Heart failure	0.108	0.108	0.00	0.2%	€1,737,532	−4,822
Number of Ablations (Excluding Index Ablation in the Cryoablation Arm)						
Twelve months	0.07	0.25	−0.18	−72.5%		
Time horizon (40 years)	0.28	1.17	−0.90	−76.5%		

*Abbreviations:* *AADs* antiarrhythmic drugs, *NNT* number needed to treat

Results also reported that the German health care system would have to spend €25,897 for each stroke event avoided in first-line PAF population, with 72 individuals needing to receive cryoablation to avoid one additional stroke event. There was little difference found in predicted lifetime rate of heart failure (0.0002) between treatment arms (Table 4).

Cryoablation remained cost-effective versus AADs in all scenarios explored at a WTP threshold of €35,000 per QALY gained. Cryoablation was cost saving and attained a dominant ICER in two scenarios explored; when a 12-week blanking period was implemented, and when health state specific stroke RR values from published literature were implemented (values available in Table S24). When the RR of AF resolution relative to the number of previous ablations was increased by 10% the highest ICER of all scenarios explored was observed (€3,965), but this was still far below the WTP threshold, as presented in Table 5.

**Table 5:** Outputs of the scenario analyses

Scenario †	Incremental Costs	Incremental QALYs	ICER
Probabilistic base case	€191	0.18	€1,037
Blanking period implemented	-€711	0.09	Dominant
Increased RR of AF recurrence relative to the number of previous ablations by 10%	€96	0.18	€537
Increased RR of AF resolution relative to the number of previous ablations by 10%	€593	0.15	€3,965
Decreased ablation success rate of 30% (proportionally)	€298	0.17	€1,767
Decreased incidence rate of stroke by 30% (proportionally)	€372	0.16	€2,351
Changed health state specific stroke RR values to values sourced from published literature	-€137	0.25	Dominant
Increased RR of developing heart failure for those in the permanent health state by 10%	€360	0.16	€2,225

*Abbreviations:* *AF* atrial fibrillation, *ICER* incremental cost-effectiveness ratio, *RR* relative risk

† All scenario analysis outputs are deterministic and incremental values are reported on a per patient basis

## Discussion

Results from the economic analysis estimated cryoablation to incur a cost of €191 per patient over a lifetime compared with AADs, while offering an increase in QALYs. Cryoablation attained an average ICER of €1,037 and had a 98.0% probability of being cost-effective at a WTP threshold of €35,000 per QALY gained. Furthermore, it was indicated through 5,000 PSA iterations that cryoablation has a 41.5% probability of being cost saving. Patients were expected to receive ~ 1.2 ablations over a lifetime, regardless of their initial treatment. However, those initially treated with cryoablation rather than AADs experienced 0.9 fewer re-ablations and a 45% reduction in time spent in AF health states. Overall, the results indicated that cryoablation would be a cost-effective alternative to AADs as an initial rhythm control therapy in PAF.

The IPD statistical analyses indicated a significant reduction in the rate of re-ablation and recurrence of AF in the cryoablation arm. Furthermore, for those receiving cryoablation in the ST-episodic health state, a predicted increase of 4.26% for 12-month utility was observed compared to those with NSR. Thus, the higher estimated QALY yield in the cryoablation arm is attributable to less time spent in health states that incur greater utility decrements. This finding is consistent with previous observations that the patient-reported decline in HRQoL is due to symptoms and AEs associated with AF [29].

A localised CEA of the German subsample (n = 1,664, 60% of the overall sample) enrolled in the Early Treatment of Atrial Fibrillation for Stroke Prevention Trial (EAST-AFNET 4) was recently conducted comparing general early rhythm control (ERC) to usual care [30]. ERC is the early application of one or more AF treatment options (including AAD, ablation, and cardioversion) with the goal of reducing the risk of AF worsening and minimizing the occurrence of AEs [30]. ERC was found to incur higher costs but resulted in a significantly longer average time to death, stroke, or hospitalization for worsening heart failure or acute coronary syndrome. Thus, ERC was deemed to be the cost-effective treatment option, reporting ICERs of €10,638 per additional year without a primary outcome and €22,536 per life year gained at a WTP threshold of ≥ €55 000. This significant finding aligns with the current study which identified that those treated with cryoablation have reduced time spent in symptomatic health states in which AEs occur. Although the current study also demonstrated a cost-effective outcome for cryoablation, the CEA of EAST-AFNET 4 did not include QALYs as a relative outcome measure for cost-effectiveness, disallowing direct comparison of cost-effectiveness through ICERs.

Although previous studies in other countries have evaluated the potential cost-effectiveness of AF ablation specifically; to the best of the authors knowledge this study is novel in providing a CEA of cryoablation versus AADs in a first-line German setting. A search into previous literature identified that the current results align with economic analyses of second-line cryoablation conducted from other European perspectives.

NICE concluded that second-line cryoablation was cost-effective compared with AADs from the perspective of the UK National Health Service (NHS) through economic evaluation [31]. An ICER of £11,687 per QALY gained was reported. Moreover, a more recent study from the perspective of the UK NHS assessed the cost-effectiveness of second-line catheter ablation versus AADs for the treatment of AF using real-world data. The findings further supported those of the current study that catheter ablation is a cost-effective treatment for atrial fibrillation compared to AADs concluding an ICER of £8,583 per QALY gained [32].

Looking more broadly, the cost-effectiveness of second-line cryoablation versus AADs was investigated in the USA through an economic analysis of the CABANA clinical trial [33]. This study found that although cryoablation was costlier due to the initial ablation procedure, a substantial enough QALY benefit was seen, offsetting the higher incremental costs and generating a cost-effective result at a WTP threshold of $100,000. This aligns with the findings of the current study and suggests that cryoablation is deemed cost-effective in both first-line and second-line settings and in different healthcare systems around the world. Therefore, our findings suggesting that cryoablation is cost-effective in a German healthcare setting is in agreement with the current literature findings.

The Institut für Qualität und Wirtschaftlichkeit im Gesundheitswesen (IQWiG) in Germany has previously supported CEA conducted through health economic modelling as a method for evaluating medical treatments [20]. As extensive details of the statistical IPD analysis (Supplementary Materials Sect. 1, Additional file 1) and the relevant RCTs [9, 10, 15] are available, and the methods and results of this CEM are explained transparently in the current paper, this study meets IQWiG criteria for the use of CEA through economic modelling for treatment investigation [20].

Although the QALY is not the preferred metric for national health technology assessment (HTA) in Germany, it does carry value with local payers for resource use allocation. For example, the third version of the Hanover Consensus recommends that, in German CEA, utility values such as QALYs are a viable outcome when based on generic instruments or TTO values [34]. Further, IQWiG noted that QALYs are acceptable measures of health benefits when the data is based upon participants of clinical trials, which aligns with the current model inputs being heavily based on RCT data [20]. The current study investigated the clinical and economic implications of implementing first-line cryoablation (Arctic Front Advance Cryoballoon) as an alternative to first-line AADs for treating symptomatic PAF from a German healthcare payer perspective.

### Strengths

There are several strengths of the current study. Firstly, the parameter estimates used in the CEM were, where possible, derived from statistical IPD analysis from three RCTs [9, 10, 15]. Secondly, the PSA and scenario analyses supported that the results were robust across all included scenarios, despite the assumptions incorporated for a number of parameters. Finally, the model structure, parameters and assumptions were validated by clinical experts.

### Limitations

Electrocardiogram (ECG) monitoring data collected during the RCTs was used to derive the CEM health state parameters. ECG monitoring detects both symptomatic and asymptomatic PAF events, meaning the rate of AF recurrence and, consequently, the re-treatment costs may be overestimated. However, consistent monitoring procedures were applied to both arms within the trials, so the presence of asymptomatic events impacted both equally. Thus, it is unlikely that treatment type would affect the proportion of events presenting as asymptomatic. To account for any impact this may have on the results, the ECG monitoring was included as a confounding effect in the regression models.

## Conclusions

Due to an associated lower rate of AEs and reduction of time in severe health states, implementing cryoballoon ablation could benefit patient HRQoL. On average the German healthcare system would also be subject to fewer costs associated with AEs and re-ablations, allowing resources to be used in other high demand areas of care (98% probability of being cost-effective). Thus, implementing this cost-effective treatment would benefit the healthcare system as a whole.

The analysis with a life-time horizon of 40-years may be useful for healthcare decision makers in Germany when considering the benefits of cryoballoon ablation over AAD in their decision-making to optimize management of patients.

At the time of publication, this economic analysis is the first to investigate the cost-effectiveness of cryoablation versus AADs for symptomatic PAF from a first-line German healthcare payer perspective. The findings illustrate that first-line cryoablation (Arctic Front Advance Cryoballoon) is a cost-effective alternative to first-line AADs as an initial rhythm control technique for patients with PAF in Germany.

## Supplementary Information

Supplementary Material 1.

## Data Availability

The datasets generated and/or analysed during the current study are not publicly available due to privacy of the individuals that participated in the study but are available from the corresponding author on reasonable request.

## Abbreviations

AE: Adverse event.
AAD: Antiarrhythmic drug
AF: Atrial fibrillation
CA: Catheter ablation
CEM: Cost-effectiveness model
CEA: Cost-effectiveness analysis
CrI: Credible intervals
EHRA: European Heart Rhythm Association
ERC: Early rhythm control
ECG: Electrocardiogram
aG-DRG: German diagnosis-related group
HRQoL: Health-related quality of life
ICER: Incremental cost-effectiveness ratio
IPD: Individual patient-level data
IQWiG: Institut für Qualität und Wirtschaftlichkeit im Gesundheitswesen
InEK: Institute for the Hospital Remuneration System
LT: Long-term
NICE: National Institute for Health and Care Excellence
NSR: Normal sinus rhythm
PAF: Paroxysmal atrial fibrillation
PSA: Probabilistic sensitivity analysis
PVI: Pulmonary vein isolation
PV: Pulmonary vein
QALY: Quality-adjusted life year
RCT: Randomized controlled trial
RR: Relative risk
ST: Short-term
TTO: Time trade-off
USA: United States of America
WTP: Willingness-to-pay
