# Supplementary material for: An economic evaluation of first-line cryoballoon ablation versus antiarrhythmic drug therapy for the treatment of paroxysmal atrial fibrillation from a German healthcare payer perspective
Source: BMC Health Serv Res. 2024 Nov 26;24:1474. doi: 10.1186/s12913-024-11967-0 (PMC11600593; doi:10.1186/s12913-024-11967-0)
Supplement: Supplementary file 1 — Supplementary Material 1. [file 12913_2024_11967_MOESM1_ESM.docx]

# An Economic Evaluation of First-line Cryoballoon Ablation Versus Antiarrhythmic Drug Therapy for the Treatment of Paroxysmal Atrial Fibrillation from a German Healthcare Payer Perspective.

Supplementary Material

*Journal*

BMC Health Services Research

*Author information*

Malte Kuniss, Lucy Hillcoat, Joe Moss, Florian Straube, Jason Andrade, Oussama Wazni, Gian Battista Chierchia, Lukas Schwegmann, Eleni Ismyrloglou, Alicia Sale, Stuart Mealing, Tom Bromilow, Emily Lane, Damian Lewis, Andreas Goette.

*Corresponding Author*

Malte Kuniss; Email address: M.Kuniss@kerckhoff-klinik.de; Kerckhoff Heart Center, Bad Nauheim, Germany.

*Caption*

This file contains details of the statistical analysis embedded in the German cost-effectiveness model as well as further details of model parameter inputs including calculations where relevant.

1. *Pooled and trial-specific baseline characteristics for all populations and the statistical analyses outputs.*

The pooled and trial-specific baseline characteristics for all populations in the IPD analyses are presented in Table S1. Patients were excluded from the analysis if they left the study <30 days following the initial procedure, or <30 days from their final ablation procedure. To control nesting effects in all statistical analyses, each clinical trial was assigned a unique Study ID. The pooled characteristics were assumed to broadly represent the general first-line PAF population in Germany. Statistical analyses were performed in R v4.1.1 [1], and missing data were assumed to be missing completely at random.

*Baseline characteristics*

Table S1: Baseline characteristics from the clinical trials

| *Characteristic* | *Cryo-FIRST* | | *STOP AF First* | | *EARLY-AF* | | *Pooled* | |
| --- | --- | --- | --- | --- | --- | --- | --- | --- |
|  | *Cryo* | *AAD* | *Cryo* | *AAD* | *Cryo* | *AAD* | *Cryo* | *AAD* |
| Patient counts [n] | 97 | 105 | 103 | 97 | 154 | 147 | 354 | 349 |
| Age (Years) [mean (sd)] | 49.9 (12.6) | 54.4 (13.5) | 60.5 (11.2) | 61.3 (11.2) | 57.8 (11.5) | 59.7 (10.5) | 56.5 (12.4) | 58.5 (12.0) |
| Sex (% Male) | 70.10% | 64.76% | 61.17% | 58.76% | 72.72% | 69.39% | 68.60% | 65.00% |
| EQ-5D-3L derived utility [mean (sd)] |  |  | 0.89 (0.19) | 0.90 (0.15) | 0.87 (0.16) | 0.87 (0.17) | 0.88 (0.17) | 0.88 (0.16) |
| EHRA Class (%) | | | | | | | | |
| I | 0% | 0% |  |  |  |  |  |  |
| II | 69.1% | 75.2% |  |  |  |  |  |  |
| III | 28.9% | 23.8% |  |  |  |  |  |  |
| IV | 2.06% | 0.6% |  |  |  |  |  |  |

Abbreviations: AAD, antiarrhythmic drugs; Cryo, cryoablation; EQ-5D-3L, EuroQol 5-Dimensions 3-Levels; EHRA, European Heart Rhythm Association.

All outcomes were defined as functions of the treatment arm. Further covariates of potential clinical relevance were utilised to produce adjusted mean estimates. Generalized linear models (GLMs) and generalized linear mixed models (GLMMs), with either a Poisson (log link), Binomial (logit link) or a Beta (logit link) distribution, were used to model all outcomes. The most appropriate distribution for all statistical models was chosen based on the diagnostic criteria (e.g., Akaike’s Information Criteria) and dependent variable type (e.g., count or continuous).

Within the long-term follow-up count-based statistical models, an offset variable was included to derive a rate per month rather than an absolute count for each patient. This was to account for exposure time for the relevant models. Utility values obtained via the EuroQol five dimensions (five levels) (EQ-5D-5L) instrument were mapped to the EQ-5D-3L by means of the van Hout crosswalk function algorithm [2]. The crosswalk function was developed based on international datasets and, thus, was deemed appropriate for a German-based value set.

Secondary statistical analysis was performed whereby outcome data collected during a 12-week blanking period were not considered. The “blanking period” was in accordance with an expert consensus statement on catheter and surgical ablation of AF, recommending the exclusion of AF recurrences within the first three months of a trial. The described analyses were conducted to assess the CEMs sensitivity to resource usage in the first 12 weeks of the clinical trial to confirm no excessive resource use disproportionately influenced the results.

The type of model, link function, offset variable and distribution used in each analysis is detailed in Supplementary Tables S2 to S10. Stepwise deletion was used for model refinement, whereby statistically non-significant covariates (p<0.05) that do not contribute to the predictive capability of the model were removed.

*Rate of AF recurrence and resolution*

A GLM with a Poisson distribution and log-link function was used to generate the rate of AF recurrence and resolution. A three-monthly rate was derived using an offset variable for the natural log of exposure time. On average, patients receiving cryoablation have a 46.7% lower three-monthly AF recurrence rate than those receiving AADs. However, there was no statistically significant treatment impact on AF resolution (p>0.05), and so stepwise deletion was used to remove this from the regression model.

Table S2: Three-monthly rate of AF recurrence (whole study period)

|  | Coefficient | Standard Error | z-value | p-value |
| --- | --- | --- | --- | --- |
| Intercept | -2.771 | 0.175 | -15.802 | <0.001* |
| Treatment (Cryo) | -0.629 | 0.130 | -4.843 | <0.001* |
| Ambulatory device (Yes) | 0.484 | 0.216 | 2.245 | 0.024* |
| Implantable loop recorder (Yes) | 1.162 | 0.186 | 6.234 | <0.001* |
| *p* * = output reached statistical significance at 95% confidence interval. | | | | |

Table S3: Three-monthly rate of AF resolution (whole study period)

|  | Coefficient | Standard Error | z-value | p-value |
| --- | --- | --- | --- | --- |
| Intercept | -0.441 | 0.094 | -4.665 | <0.001* |
| Implantable loop recorder (Yes) | 0.403 | 0.124 | 3.259 | 0.001* |
| *p* * = output reached statistical significance at 95% confidence interval. | | | | |

*Rate of repeat ablation (re-ablation)*

The rate of repeat ablation was derived using a GLM with a Poisson distribution and log-link function. A monthly rate was derived using an offset variable for the natural log of exposure time. A significant treatment effect was reported for the monthly rate of re-ablation (p<0.001) (Table S4), with those receiving cryoablation experiencing an average rate of re-ablation that is 72.5% lower than those receiving AADs within the German cost-effectiveness model (CEM) over 12 months.

Table S4: Rate of re-ablation (whole study period)

|  | Coefficient | Standard Error | z-value | p-value |
| --- | --- | --- | --- | --- |
| Intercept | -3.843 | 0.108 | -35.639 | <0.001* |
| Treatment (Cryo) | -1.302 | 0.231 | -5.640 | <0.001* |
| *p* * = output reached statistical significance at 95% confidence interval. | | | | |

*EQ-5D-3L utility values*

A GLMM with a Beta distribution and a logit link function was used to generate utility values. To control for variation between patients, a random effect was included. After stepwise selection, utility at baseline (p<0.001) and the treatment arm (p=0.025) were maintained as the only statistically significant predictors of utility at 12 months (Table S5a). Patients with ST episodic AF and those in the NSR health state were not found to be significantly different (p=0.115). Table S5b shows the utility values used for the NSR and ST episodic health states stratified by treatment arm using the deterministic model inputs. The estimated decrement between health states was applied to the population norm utility values.

Table S5a: Twelve-month EQ-5D-3L utility

|  | Estimate | Standard Error | z-value | *p*-value |
| --- | --- | --- | --- | --- |
| Intercept | -0.282 | 0.260 | -1.084 | 0.278 |
| AF status (ST-AF) | -0.747 | 0.474 | -1.576 | 0.115 |
| Treatment (Cryo) | 0.219 | 0.098 | 2.234 | 0.025* |
| Baseline utility | 2.689 | 0.289 | 9.319 | <0.001* |
| *p* * = output reached statistical significance at 95% confidence interval. | | | | |

Table S5b: Estimated utility values used in the cost-effectiveness model using the regression equation and determinist inputs

| Health State | Cryoablation | AADs |
| --- | --- | --- |
| NSR | 0.91 | 0.89 |
| ST Episodic | 0.83 | 0.79 |
| Decrement | 0.08 | 0.10 |

Abbreviations: NSR, normal sinus rhythm; ST, short-term

*Cardiovascular-related hospitalization, accident and emergency visits, pharmaceutical and electrical cardioversion*

A GLMM with a Poisson distribution and log-link function was used to derive the rates of cardiovascular-related hospitalisation, emergency department visits and cardioversion. A monthly rate was produced using an offset variable for time. A random effect was included to control for variation between patients. A statistically significant treatment effect was found for the monthly rate of electrical (p=0.021) and pharmaceutical cardioversion (p<0.001) (Tables S8 and S9). More specifically, patients receiving cryoablation reported a 48.9% and 82.5% average reduction for a monthly rate of electrical and pharmaceutical cardioversion, respectively, compared with those receiving AADs.

Table S6: Rate of cardiovascular-related hospitalization (whole study period)

|  | Coefficient | Standard Error | z-value | *p*-value |
| --- | --- | --- | --- | --- |
| Intercept | -9.235 | 0.694 | -13.307 | <0.001* |
| *p* * = output reached statistical significance at 95% confidence interval. | | | | |

Table S7: Rate of cardiovascular-related accident and emergency visits (whole study period)

|  | Coefficient | Standard Error | z-value | *p*-value |
| --- | --- | --- | --- | --- |
| Intercept | -2.978 | 0.283 | -10.519 | <0.001* |
| *p* * = output reached statistical significance at 95% confidence interval. | | | | |

Table S8: Rate of electrical cardioversion (whole study period)

|  | Coefficient | Standard Error | z-value | p-value |
| --- | --- | --- | --- | --- |
| Intercept | -4.815 | 0.171 | -28.074 | <0.001* |
| Treatment (Cryo) | -0.672 | 0.291 | -2.304 | 0.021* |
| *p* * = output reached statistical significance at 95% confidence interval. | | | | |

Table S9: Rate of pharmaceutical cardioversion (whole study period)

|  | Coefficient | Standard Error | z-value | *p*-value |
| --- | --- | --- | --- | --- |
| Intercept | -4.234 | 0.864 | -4.898 | <0.001* |
| Treatment (Cryo) | -1.744 | 0.489 | -3.566 | <0.001* |
| Age | -0.036 | 0.013 | -2.852 | 0.004* |
| 7-day Holter | 1.574 | 0.538 | 2.923 | 0.003* |
| *p* * = output reached statistical significance at 95% confidence interval. | | | | |

*Rate of Outpatient Visits*

A GLM with a Poisson distribution and a log-link function was used to derive the rate of cardiovascular-related outpatient appointments. To control for variation between patients, a random effect was included. A monthly rate was produced using an offset variable for time.

Table S10: Rate of cardiovascular-related outpatient appointments (whole study period)

|  | Estimate | Standard Error | z-value | p-value |
| --- | --- | --- | --- | --- |
| Intercept | -9.143 | 0.650 | -14.065 | <0.001* |
| *p* * = output reached statistical significance at 95% confidence interval. | | | | |

1. *Transition probabilities*

Transition probabilities used for calculating the probability of moving between health states (excluding death) per cycle were derived using the previously outlined statistical equations. The probabilities are presented for both model arms in Table S11 and Table S12.

Table S11: State transition summary table for cryoablation arm

| **From\To** | **NSR 0** | **NSR 1** | **NSR 2** | **ST Epi 0** | **ST Epi 1** | **ST Epi 2** | **LT Per 0** | **LT Per 1** | **LT Per 2** | **Perm** |
| --- | --- | --- | --- | --- | --- | --- | --- | --- | --- | --- |
| **NSR 0** | 0.939 |  |  | 0.061 |  |  |  |  |  |  |
| **NSR 1** |  | 0.939 |  |  | 0.061 |  |  |  |  |  |
| **NSR 2** |  |  | 0.930 |  |  | 0.070 |  |  |  |  |
| **ST Epi 0** | 0.53 | 0.01 |  | 0.410 | 0.00 |  | 0.039 |  |  |  |
| **ST Epi 1** |  | 0.53 | 0.01 |  | 0.41 | 0.00 |  | 0.04 |  |  |
| **ST Epi 2** |  |  | 0.45 |  |  | 0.50 |  |  | 0.04 |  |
| **LT Per 0** | 0.14 | 0.03 |  | 0.07 |  |  | 0.74 | 0.01 |  | 0.02 |
| **LT Per 1** |  | 0.14 | 0.03 |  | 0.07 |  |  | 0.74 | 0.01 | 0.02 |
| **LT Per 2** |  |  | 0.12 |  |  | 0.06 |  |  | 0.80 | 0.02 |
| **Perm** |  |  |  |  |  |  |  |  |  | 1.00 |

The number following health state descriptions details the number of re-ablation procedures.

Abbreviations: Epi, episodic; LT, long-term; NSR, normal sinus rhythm; Perm, permanent; ST, short-term.

Table S12: State transition summary table for AADs arm

| **From\To** | **NSR 0** | **NSR 1** | **NSR 2** | **NSR 3** | **ST Epi 0** | **ST Epi 1** | **ST Epi 2** | **ST Epi 3** | **LT Per 0** | **LT Per 1** | **LT Per 2** | **LT Per 3** | **Perm** |
| --- | --- | --- | --- | --- | --- | --- | --- | --- | --- | --- | --- | --- | --- |
| **NSR 0** | 0.89 |  |  |  | 0.11 |  |  |  |  |  |  |  |  |
| **NSR 1** |  | 0.89 |  |  |  | 0.11 |  |  |  |  |  |  |  |
| **NSR 2** |  |  | 0.87 |  |  |  | 0.13 |  |  |  |  |  |  |
| **NSR 3** |  |  |  | 0.85 |  |  |  | 0.15 |  |  |  |  |  |
| **ST Epi 0** | 0.53 | 0.05 |  |  | 0.36 | 0.02 |  |  | 0.0385 |  |  |  |  |
| **ST Epi 1** |  | 0.53 | 0.05 |  |  | 0.36 | 0.02 |  |  | 0.04 |  |  |  |
| **ST Epi 2** |  |  | 0.45 | 0.05 |  |  | 0.44 | 0.02 |  |  | 0.04 |  |  |
| **ST Epi 3** |  |  |  | 0.39 |  |  |  | 0.56 |  |  |  | 0.05 |  |
| **LT Per 0** | 0.14 | 0.03 |  |  | 0.07 |  |  |  | 0.74 | 0.01 |  |  | 0.02 |
| **LT Per 1** |  | 0.14 | 0.03 |  |  | 0.07 |  |  |  | 0.74 | 0.01 |  | 0.02 |
| **LT Per 2** |  |  | 0.12 | 0.03 |  |  | 0.06 |  |  |  | 0.76 | 0.01 | 0.02 |
| **LT Per 3** |  |  |  | 0.10 |  |  |  | 0.05 |  |  |  | 0.83 | 0.02 |
| **Perm** |  |  |  |  |  |  |  |  |  |  |  |  | 1.00 |

The number following health state descriptions details the number of re-ablation procedures.

Abbreviations: Epi, episodic; LT, long-term; NSR, normal sinus rhythm; Perm, permanent; ST, short-term.

1. *Breakdown of calculated costs*

**Ablation procedure costs**

The total ablation cost applied in the model was calculated using a weighted average of 2022 DRG costs for inpatient ablation (including bedside nursing), and proportional short, normal and long length of stay values from InEK DatenBrowser 2021 [3]. Procedural code 8-835.a5 for cryoablation was used to filter ablation cases. An assumption was made for the number of days associated with each length of stay (LoS) to calculate the related nursing costs from a payer perspective. DRGs were calculated using the federal DRG base rate 2023 of €4,000 [4]. Bedside nursing was calculated using nursing fee value.

Table S13: Ablation procedure unit cost

| DRG cost (including bedside nursing) | Assumption for inpatient days (LoS type) | Proportion of each LoS type | Weighted cost |
| --- | --- | --- | --- |
| €7,394.20 | 1 day (short) | 28.34% | €2,392 |
| €8,324.40 | 2 days (normal) | 63.96% | €5,085 |
| €9,841.60 | 8 days (long) | 7.70% | €643 |
| Weighted Average | | | €8,121 |
| Abbreviations: LoS, length of stay. | | | |

**Pharmaceutical costs (per cycle)**

The pharmaceutical costs per cycle were applied in the model for all relevant drugs. The doses were sourced from the British National Formulary (BNF), with other values presented in Table 14a sourced from the 2023 Rote Liste [5, 6]. Table 14b presents the overall cost per treatment arm, calculated from the cost per cycle weighted by the number of patients on each drug, divided by the total number of patients in the treatment arm.

| **Type of Drug** | **Drug** | **mg Per Day** | **Pharmacy Retail Price** | **Patient Co-payment** | **mg Per Tablet** | **Pack Size (tablets)** | **Cost Per mg** | **Total mg Per Year** | **Cost Per Cycle** |
| --- | --- | --- | --- | --- | --- | --- | --- | --- | --- |
| Anti-coagulation at enrolment | Warfarin | 7.5 | €23.19 | 9.86 | 5 | 100 | 0.03 € | 2,738 | €18.25 |
|  | Aspirin | 75 | €6.25 | 0.00 | 75 | 100 | 0.00 € | 27,375 | €5.70 |
|  | Rivaroxaban | 20 | €321.04 | 0.00 | 20 | 98 | 0.16 € | 7,300 | €298.93 |
|  | Dabigatran | 220 | €113.63 | 0.00 | 110 | 180 | 0.01 € | 80,300 | €115.21 |
|  | Apixaban | 10 | €343.39 | 0.00 | 5.0 | 200 | 0.34 € | 3,650 | €313.34 |
|  | Edoxaban | 45 | €249.55 | 0.00 | 60 | 98 | 0.04 € | 16,425 | €174.27 |
|  | Phenprocoumon | 3 | €18.28 | 0.00 | 3 | 100 | 0.06 € | 2,190 | €33.36 |
|  | Ticagrelor | 180 | €105.34 | 0.00 | 60 | 60 | 0.03 € | 65,700 | €480.61 |
| Class I/III Anti-Arrhythmics | Amiodarone | 200 | €35.21 | 5.00 | 100 | 100 | 0.00 € | 77,200 | €58.31 |
|  | Dronedarone | 800 | €131.86 | 10.00 | 400 | 100 | 0.00 € | 292,000 | €222.39 |
|  | Flecainide | 100 | €51.03 | 5.10 | 100 | 100 | 0.00 € | 36,500 | €41.91 |
|  | Propafenone | 450 | €17.60 | 0.00 | 150 | 100 | 0.00 € | 164,250 | €48.18 |
|  | Sotalol | 240 | €16.61 | 0.00 | 40 | 100 | 0.00 € | 87,600 | €90.94 |

Table S14a: Pharmaceutical costs (per cycle)

Table S14b: Total pharmaceutical cost per intervention (per cycle)

| **Intervention** | **Total Cost Per Cycle** |
| --- | --- |
| Cryoablation | €69 |
| AADs | €89 |

1. *Adverse event parameters*

Table S15: Stroke risk by CHA₂DS₂-VASc score

| Score | Risk | Source |
| --- | --- | --- |
| 0 | 0.2% | [7] |
| 1 | 0.6% |  |
| 2 | 2.2% |  |
| 3 | 3.2% |  |
| 4 | 4.8% |  |
| 5 | 7.2% |  |
| 6 | 9.7% |  |
| 7 | 11.2% |  |
| 8 | 10.8% |  |
| 9 | 12.2% |  |

Table S16: CHA₂DS₂-VASc score by age

| Age category | Score | Source |
| --- | --- | --- |
| 15 to 39 | 1.3 | Baseline study data.  Those aged 60 - 79 have their CHA₂DS₂-VASc score increased by 1.  Those aged 80+ have their CHA₂DS₂-VASc score increased by 2. |
| 40 to 49 | 1.3 |  |
| 50 to 59 | 1.3 |  |
| 60 to 69 | 2.3 |  |
| 70 to 79 | 2.3 |  |
| 80 to 89 | 3.3 |  |
| 90 and over | 3.3 |  |

Table S17: Stroke incidence by age and CHA₂DS₂-VASc score

| Age category | Deterministic |
| --- | --- |
| 15 to 39 | 1.1% |
| 40 to 49 | 1.1% |
| 50 to 59 | 1.1% |
| 60 to 69 | 2.5% |
| 70 to 79 | 2.5% |
| 80 to 89 | 3.7% |
| 90 and over | 3.7% |

Table S18: Treatment and health state-specific stroke relative risk values (used to adjust the general AF population stroke rates derived from study population CHA2-DS2-VASc scores)

| Values used in the base case | | | |
| --- | --- | --- | --- |
| Health state | AADs | Cryoablation | Source |
| NSR versus general AF population | 0.34 | 0.34 | Clinical Assumption |
| ST-Episodic versus general AF population | 0.40 | 0.40 |  |
| LT-Persistent versus general AF population | 0.60 | 0.60 |  |
| Permanent versus general AF population | 1.50 | 1.50 |  |

Table S19: Heart failure incidence by age in the general population

| Age category | Deterministic | Source |
| --- | --- | --- |
| 15 to 34 | 0.004% | [8] |
| 35 to 44 | 0.013% |  |
| 45 to 54 | 0.050% |  |
| 55 to 64 | 0.200% |  |
| 65 to 74 | 0.630% |  |
| 75+ | 1.640% |  |

Table S20: Heart failure severity distribution

| NYHA class | Share | Source |
| --- | --- | --- |
| I | 22.14% | [9] |
| II | 40.52% |  |
| III | 28.99% |  |
| IV | 8.34% |  |

1. *Mortality parameters*

The formula detailed below was used for deriving the health state-specific overall annual mortality rates (include stroke and heart failure mortality).

*All-cause mortality = [(baseline annual mortality rate [excl. stroke and HF] + (baseline annual stroke mortality rate * probability of stroke) + (baseline annual HF mortality rate * probability of HF)]*

The overall annual mortality rates were then converted to a three-monthly probability to align with the cycle length and for application in the model using the following formula:

*1-exp(-rate*(cycle length/12))*

Table S21: General mortality rates

| Age | Male | Female | Overall | Source |
| --- | --- | --- | --- | --- |
| 15 - 19 | 0.03% | 0.01% | 0.03% | [10]  Mortality excluding stroke and heart failure (annual) was defined as all-cause mortality excl. ICD-10 I60-69 (stroke mortality) and I30-I52 (heart failure mortality). |
| 20 - 24 | 0.04% | 0.02% | 0.03% |  |
| 25 - 29 | 0.05% | 0.02% | 0.04% |  |
| 30 - 34 | 0.06% | 0.03% | 0.05% |  |
| 35 - 39 | 0.10% | 0.05% | 0.08% |  |
| 40 - 44 | 0.15% | 0.08% | 0.12% |  |
| 45 - 49 | 0.25% | 0.14% | 0.21% |  |
| 50 - 54 | 0.41% | 0.22% | 0.35% |  |
| 55 - 59 | 0.68% | 0.36% | 0.58% |  |
| 60 - 64 | 1.13% | 0.58% | 0.95% |  |
| 65 - 69 | 1.78% | 0.93% | 1.50% |  |
| 70 - 74 | 2.54% | 1.40% | 2.16% |  |
| 75 - 79 | 4.11% | 2.48% | 3.57% |  |
| 80 - 84 | 6.27% | 4.07% | 5.54% |  |
| 85 - 89 | 10.81% | 7.90% | 9.85% |  |
| 90 - 94 | 21.46% | 18.26% | 20.40% |  |
| 95 - 99 | 0.03% | 0.01% | 0.03% |  |
| 100+ | 0.04% | 0.02% | 0.03% |  |

Table S22: Stroke mortality rates

| Age category | Mortality rate | Source |
| --- | --- | --- |
| 18 to 24 | 3.90% | [11] |
| 25 to 34 | 3.90% |  |
| 35 to 44 | 3.90% |  |
| 45 to 54 | 3.90% |  |
| 55 to 64 | 6.20% |  |
| 65 to 74 | 10.65% |  |
| 75+ | 19.00% |  |

Table S23: Heart failure mortality rates

| Age category | Mortality rate | Source |
| --- | --- | --- |
| 16 to 24 | 16.44% | [12] |
| 25 to 34 | 16.44% |  |
| 35 to 44 | 16.44% |  |
| 45 to 54 | 16.44% |  |
| 55 to 64 | 20.39% |  |
| 65 to 74 | 29.65% |  |
| 75+ | 47.05% |  |

1. *Health state specific stroke relative risk values*

Table S24: Health state specific stroke relative risk values used in scenario analysis

Table S24 presents the relative risk values that replaced the base case values (presented in table S18) within the scenario analysis. This was to test the uncertainty of the relative risk values, measured by how much replacing them impacted the model results.

| Health state | AADs | Cryoablation | Source |
| --- | --- | --- | --- |
| NSR versus general population | 1.00 | 1.00 | Assumption |
| ST-Episodic versus general population | 2.12 | 2.12 | [13] |
| LT-Persistent versus ST-Episodic | 1.44 | 1.44 | [14] |
| Permanent versus ST-Episodic | 1.83 | 1.83 |  |

1. *CHEERS 2022 Checklist [15]*

| **Topic** | **No.** | **Item** | **Location where item is reported** |
| --- | --- | --- | --- |
| **Title** |  |  |  |
|  | 1 | Identify the study as an economic evaluation and specify the interventions being compared. | Title, Page 1 |
| **Abstract** |  |  |  |
|  | 2 | Provide a structured summary that highlights context, key methods, results, and alternative analyses. | Abstract, Page 2 - 3 |
| **Introduction** |  |  |  |
| **Background and objectives** | 3 | Give the context for the study, the study question, and its practical relevance for decision making in policy or practice. | Background, Page 3 - 4 |
| **Methods** |  |  |  |
| **Health economic analysis plan** | 4 | Indicate whether a health economic analysis plan was developed and where available. | Methods, Page 4 – 10. Note: The model is not currently publicly available. |
| **Study population** | 5 | Describe characteristics of the study population (such as age range, demographics, socioeconomic, or clinical characteristics). | Methods, Page 4 |
| **Setting and location** | 6 | Provide relevant contextual information that may influence findings. | Methods, Page 4 - Link to the underlying clinical study |
| **Comparators** | 7 | Describe the interventions or strategies being compared and why chosen. | Background, Page 4 |
| **Perspective** | 8 | State the perspective(s) adopted by the study and why chosen. | Methods, Page 4 - 5 |
| **Time horizon** | 9 | State the time horizon for the study and why appropriate. | Methods, Page 5 |
| **Discount rate** | 10 | Report the discount rate(s) and reason chosen. | Methods, Page 5 |
| **Selection of outcomes** | 11 | Describe what outcomes were used as the measure(s) of benefit(s) and harm(s). | Methods, Page 5 |
| **Measurement of outcomes** | 12 | Describe how outcomes used to capture benefit(s) and harm(s) were measured. | Methods, Page 8 |
| **Valuation of outcomes** | 13 | Describe the population and methods used to measure and value outcomes. | Methods, Page 8 |
| **Measurement and valuation of resources and costs** | 14 | Describe how costs were valued. | Methods, Page 8 and Appendix, Table 1 |
| **Currency, price date, and conversion** | 15 | Report the dates of the estimated resource quantities and unit costs, plus the currency and year of conversion. | Methods, Page 8 |
| **Rationale and description of model** | 16 | If modelling is used, describe in detail and why used. Report if the model is publicly available and where it can be accessed. | Methods, Page 4 - 9 |
| **Analytics and assumptions** | 17 | Describe any methods for analysing or statistically transforming data, any extrapolation methods, and approaches for validating any model used. | Methods, Page 6 and 9 - 10 |
| **Characterising heterogeneity** | 18 | Describe any methods used for estimating how the results of the study vary for subgroups. | N/A - No subgroup analyses considered in the study |
| **Characterising distributional effects** | 19 | Describe how impacts are distributed across different individuals or adjustments made to reflect priority populations. | N/A |
| **Characterising uncertainty** | 20 | Describe methods to characterise any sources of uncertainty in the analysis. | Methods, Page 9 - 10 and Table 5 |
| **Approach to engagement with patients and others affected by the study** | 21 | Describe any approaches to engage patients or service recipients, the general public, communities, or stakeholders (such as clinicians or payers) in the design of the study. | Methods, Page 5 |
| **Results** |  |  |  |
| **Study parameters** | 22 | Report all analytic inputs (such as values, ranges, references) including uncertainty or distributional assumptions. | Appendix, Table 1 and All Supp Tables |
| **Summary of main results** | 23 | Report the mean values for the main categories of costs and outcomes of interest and summarise them in the most appropriate overall measure. | Appendix, Table 2 |
| **Effect of uncertainty** | 24 | Describe how uncertainty about analytic judgments, inputs, or projections affect findings. Report the effect of choice of discount rate and time horizon, if applicable. | Results, Page 10 - 12 |
| **Effect of engagement with patients and others affected by the study** | 25 | Report on any difference patient/service recipient, general public, community, or stakeholder involvement made to the approach or findings of the study | Methods, Page 2 and 6 |
| **Discussion** |  |  |  |
| **Study findings, limitations, generalisability, and current knowledge** | 26 | Report key findings, limitations, ethical or equity considerations not captured, and how these could affect patients, policy, or practice. | Discussion, Page 13 to 16 |
| **Other relevant information** |  |  |  |
| **Source of funding** | 27 | Describe how the study was funded and any role of the funder in the identification, design, conduct, and reporting of the analysis | Statements and Declarations, Page 19 |
| **Conflicts of interest** | 28 | Report authors conflicts of interest according to journal or International Committee of Medical Journal Editors requirements. | Statements and Declarations, Page 18 - 19 |

# References

1. R Core Team. R: A Language and Environment for Statistical Computing. R Foundation for Statistical Computing. 2022. Available from: <https://www.R-project.org>.

2. Van Hout B, Janssen M, Feng Y-S, Kohlmann T, Busschbach J, Golicki D*, et al.* Interim scoring for the EQ-5D-5L: mapping the EQ-5D-5L to EQ-5D-3L value sets. Value in health. 2012.15(5):708-15. doi: <https://doi.org/10.1016/j.jval.2012.02.008>

3. InEK. InEK DatenBrowser. In. <https://datenbrowser.inek.org/>; 2021. Accessed 16 February 2023.

4. DRG Research Group. Grouper. In. <https://www.drg-research-group.de/>; 2022. Accessed 16 February 2023.

5. NICE. BNF: Drugs A to Z. In. <https://bnf.nice.org.uk/drugs/>; 2023

6. Liste R. In. <https://www.rote-liste.de/>; 2023

7. Friberg L, Rosenqvist M, Lip GY. Evaluation of risk stratification schemes for ischaemic stroke and bleeding in 182 678 patients with atrial fibrillation: the Swedish Atrial Fibrillation cohort study. Eur Heart J. 2012.33(12):1500-10. doi: 10.1093/eurheartj/ehr488

8. Christiansen MN, Kober L, Weeke P, Vasan RS, Jeppesen JL, Smith JG*, et al.* Age-Specific Trends in Incidence, Mortality, and Comorbidities of Heart Failure in Denmark, 1995 to 2012. Circulation. 2017.135(13):1214-23. doi: 10.1161/CIRCULATIONAHA.116.025941

9. Zhang R, Ma S, Shanahan L, Munroe J, Horn S, Speedie S. Discovering and identifying New York heart association classification from electronic health records. BMC Med Inform Decis Mak. 2018.18(Suppl 2):48. doi: 10.1186/s12911-018-0625-7

10. Destatis. Todesursachenstatistik, Todesursachen in Deutschland. In. <https://www.destatis.de/DE/Methoden/Qualitaet/Qualitaetsberichte/Gesundheit/todesursachen.pdf?__blob=publicationFile>; 2020

11. Saposnik G, Cote R, Phillips S, Gubitz G, Bayer N, Minuk J*, et al.* Stroke outcome in those over 80: a multicenter cohort study across Canada. Stroke. 2008.39(8):2310-7. doi: 10.1161/STROKEAHA.107.511402

12. Vaartjes I, Hoes AW, Reitsma JB, de Bruin A, Grobbee DE, Mosterd A*, et al.* Age- and gender-specific risk of death after first hospitalization for heart failure. BMC Public Health. 2010.10:637. doi: 10.1186/1471-2458-10-637

13. Friberg L, Hammar N, Rosenqvist M. Stroke in paroxysmal atrial fibrillation: report from the Stockholm Cohort of Atrial Fibrillation. Eur Heart J. 2010.31(8):967-75. doi: 10.1093/eurheartj/ehn599

14. Vanassche T, Lauw MN, Eikelboom JW, Healey JS, Hart RG, Alings M*, et al.* Risk of ischaemic stroke according to pattern of atrial fibrillation: analysis of 6563 aspirin-treated patients in ACTIVE-A and AVERROES. Eur Heart J. 2015.36(5):281-7a. doi: 10.1093/eurheartj/ehu307

15. Husereau D DM, Augustovski F,. Consolidated Health Economic Evaluation Reporting Standards 2022 (CHEERS 2022) Explanation and Elaboration: A Report of the ISPOR CHEERS II Good Practices Task Force. <https://don-husereau.shinyapps.io/CHEERS/>: 2022.
